# Erratum Vol. 5, No. 3

**DOI:** 10.3201/eid0505.990524

**Published:** 1999


### The 10th International Symposium on Viral Hepatitis and Liver Disease
**Atlanta, Georgia, USA, April 9–13, 2000**

Topics will include virology, epidemiology, diagnosis, treatment, and prevention. For more information contact: Organizing Secretariat, MediTech Media Ltd., Tower Place 100, 3340 Peachtree Road, Suite 550, Atlanta, GA 30326, USA; telephone: 404-233-4490; fax: 404-233-7464; e-mail: info@Hep2000.com.

**Erratum Vol. 5, No. 3**

In the article "Bacterial Vaccines and Serotype Replacement: Lessons from *Haemophilus influenzae* and Prospects for *Streptococcus pneumoniae*," by Marc Lipsitch, there is an error in the figure legend on page 339. Controls are incorrectly identified as black bars; however, in the text and the figure itself, controls are correctly represented by white bars and vaccine recipients by black bars. We regret any confusion this error may have caused.

### ICEID 2000

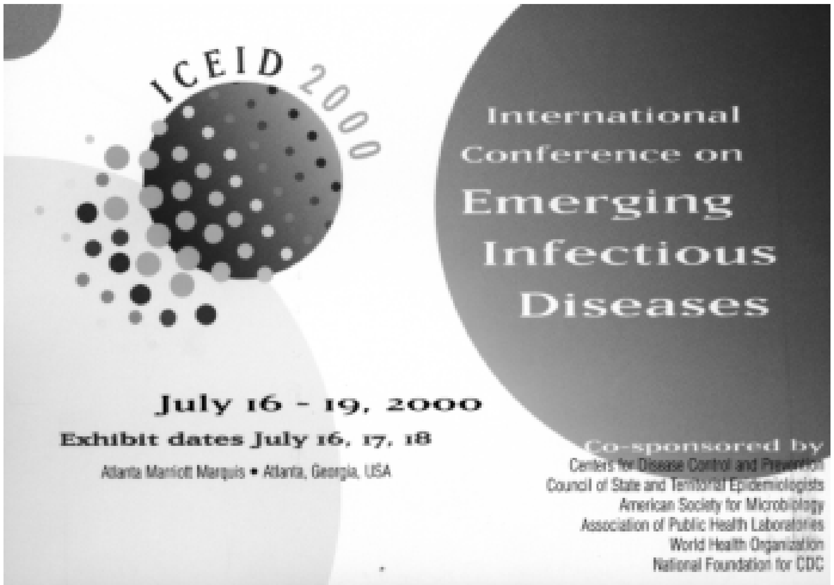

Hold the dates of July 16–19, 2000, for the International Conference on Emerging Infectious Diseases, a meeting of 2,500 specialists in infectious diseases. The program will include plenary sessions and symposia with invited speakers, presentations on emerging infections activities, and oral and poster presentations. Major topics will include current work on surveillance, epidemiology, research, communication and training, as well as prevention and control of emerging infectious diseases, both in the United States and abroad. Abstracts are invited and will be accepted beginning in October 1999.

The Call for Abstracts and Preliminary Program will be mailed in October 1999.

For more information, call ICEID management at 202-942-9248, e-mail meetinginfo@asmusa.org, or www.cdc.gov/ncidod/iced2k.htm.

In the article "Bacterial Vaccines and Serotype Replacement: Lessons from *Haemophilus influenzae* and Prospects for *Streptococcus pneumoniae*," by Marc Lipsitch, there is an error in the figure legend on page 339. Controls are incorrectly identified as black bars; however, in the text and the figure itself, controls are correctly represented by white bars and vaccine recipients by black bars. We regret any confusion this error may have caused.

